# Differential effects of buffer pH on Ca^2+^-induced ROS emission with inhibited mitochondrial complexes I and III

**DOI:** 10.3389/fphys.2015.00058

**Published:** 2015-03-10

**Authors:** Daniel P. Lindsay, Amadou K. S. Camara, David F. Stowe, Ryan Lubbe, Mohammed Aldakkak

**Affiliations:** ^1^Department of Anesthesiology, The Medical College of WisconsinMilwaukee, WI, USA; ^2^Cardiovascular Research Center, The Medical College of WisconsinMilwaukee, WI, USA; ^3^Department of Physiology, The Medical College of WisconsinMilwaukee, WI, USA; ^4^Department of Anesthesiology, VA Medical Center Research ServiceMilwaukee, WI, USA; ^5^Department of Biomedical Engineering, Marquette UniversityMilwaukee, WI, USA

**Keywords:** mitochondrial complex I, mitochondrial complex III, reactive oxygen species, simulated ischemia, mitochondrial permeability transition pore, Ca^2+^, pH

## Abstract

Excessive mitochondrial reactive oxygen species (ROS) emission is a critical component in the etiology of ischemic injury. Complex I and complex III of the electron transport chain are considered the primary sources of ROS emission during cardiac ischemia and reperfusion (IR) injury. Several factors modulate ischemic ROS emission, such as an increase in extra-matrix Ca^2+^, a decrease in extra-matrix pH, and a change in substrate utilization. Here we examined the combined effects of these factors on ROS emission from respiratory complexes I and III under conditions of simulated IR injury. Guinea pig heart mitochondria were suspended in experimental buffer at a given pH and incubated with or without CaCl_2_. Mitochondria were then treated with either pyruvate, a complex I substrate, followed by rotenone, a complex I inhibitor, or succinate, a complex II substrate, followed by antimycin A, a complex III inhibitor. H_2_O_2_ release rate and matrix volume were compared with and without adding CaCl_2_ and at pH 7.15, 6.9, or 6.5 with pyruvate + rotenone or succinate + antimycin A to simulate conditions that may occur during *in vivo* cardiac IR injury. We found a large increase in H_2_O_2_ release with high [CaCl_2_] and pyruvate + rotenone at pH 6.9, but not at pHs 7.15 or 6.5. Large increases in H_2_O_2_ release rate also occurred at each pH with high [CaCl_2_] and succinate + antimycin A, with the highest levels observed at pH 7.15. The increases in H_2_O_2_ release were associated with significant mitochondrial swelling, and both H_2_O_2_ release and swelling were abolished by cyclosporine A, a desensitizer of the mitochondrial permeability transition pore (mPTP). These results indicate that ROS production by complex I and by complex III is differently affected by buffer pH and Ca^2+^ loading with mPTP opening. The study suggests that changes in the levels of cytosolic Ca^2+^ and pH during IR alter the relative amounts of ROS produced at mitochondrial respiratory complex I and complex III.

## Introduction

Ischemic injury is a multifactorial process that predisposes to further injury during reperfusion (Camara et al., 2007; Aldakkak et al., 2008a,b, 2011). One key aspect of ischemic injury is the increase in mitochondrial production of reactive oxygen species (ROS) above the antioxidant ability of the endogenous ROS scavenging system (Trachootham et al., 2008; Stowe and Camara, 2009; Camara et al., 2010). Under normal conditions, ROS emission is maintained at a low level, which is important for regular cellular function. But an increase in ROS emission (more production and less scavenging) during ischemia leads to oxidative stress and apoptosis that leads to cellular dysfunction and death (Trachootham et al., 2008).

It is widely acknowledged that the main sources of ROS production (mainly superoxide, O^•−^_2_) during cardiac ischemia and reperfusion (IR) injury are respiratory complex I and complex III of the mitochondrial electron transport chain (ETC) (Chen et al., 2007, 2008; Kim et al., 2012; Musatov and Robinson, 2012); but it is unclear what is the relative contribution of complexes I and III on enhancing ROS generation during IR and when ROS scavenging systems fail. Unlike the other components of the ETC, these complexes are more prone to electron leak, even under physiologic conditions due to their electron transfer mechanisms (Musatov and Robinson, 2012). Thus, during IR, these complexes are prone to self-induced oxidative damage (Gadicherla et al., 2012), which impairs their activity, predisposing them to even greater ROS production (Rouslin, 1983; Chen et al., 2007, 2008; Musatov and Robinson, 2012). The damage to complex I may occur abruptly within 20 min of ischemia, whereas damage to complex III may occur more gradually as ischemia proceeds (Rouslin, 1983; Chen et al., 2007).

We showed previously in a guinea pig model of *ex vivo* global IR injury two distinct time-dependent phases of ROS emission during ischemia; an early phase of low/moderate accumulation of ROS, and a late phase of high ROS accumulation, followed by a surge of ROS during early reperfusion (Kevin et al., 2003; Camara et al., 2007; Aldakkak et al., 2008a,b, 2011). This two-phase release of ROS may correspond with the timing of damage of complexes I and III as reported by others (Chen et al., 2007). To understand this, we investigated in a recent isolated mitochondrial study the impact of extreme conditions that might mimic the period of ischemia and reperfusion on ROS emission (Aldakkak et al., 2013). We found a large increase in hydrogen peroxide (H_2_O_2_) release when complex III electron transfer was blocked by antimycin A (AA) in succinate-energized mitochondria incubated in elevated extra-matrix Ca^2+^ buffer. However, these studies did not evaluate the impact of changes in buffer pH and mitochondrial permeability transition pore (mPTP) opening by excess Ca^2+^ overload, both of which are important modulating factors that can occur in mitochondria during IR and contribute to ROS production.

During cardiac ischemia, cytosolic pH levels decrease (Park et al., 1999), due in part to increased lactate production via anaerobic glycolysis, and cytosolic Ca^2+^ levels rise (Aldakkak et al., 2011), due in part to reduced Ca^2+^ sequestration by the sarcoplasmic reticulum. Mitochondrial Ca^2+^ levels increase (Aldakkak et al., 2008a); in part as a result of the increase in uptake by the mitochondrial Ca^2+^ uniporter. Matrix pH levels, however, depend on many factors including cytosolic pH, mitochondrial Na^+^/H^+^, K^+^/H^+^, and Na^+^/Ca^2+^ exchange, proton (H^+^) leak, and variable H^+^ pumping rates by complexes I, III, IV, and V. In the first 5 min of cardiac ischemia, cytosolic pH in affected cardiomyocytes has been reported to drop 0.5 pH units from the initial pH around 7.15, and eventually to reach as low as 6.0 with a longer ischemia time (Stamm et al., 2003; Murphy and Steenbergen, 2008). Interestingly, one study (Selivanov et al., 2008) demonstrated that pH can directly modulate ROS production from the ETC; they reported that an alkaline pH increased formation of O^•−^_2_ due to increased stabilization of the semiquinone radical in the Q cycle of complex III (Selivanov et al., 2008). However, as cardiac ischemia progresses there is a gradual decrease in cellular pH (Park et al., 1999) and a gradual increase in ROS levels (Vanden Hoek et al., 1997; Becker et al., 1999; Kevin et al., 2003), indicating that factors other than just pH alone are involved.

The aim of our study was to investigate the combined effects of pH and elevated Ca^2+^ on the rate of release of H_2_O_2_ from mitochondria, using substrates and inhibitors of respiratory complexes, in an attempt to mimic cardiac IR. Specifically, we looked at the effect of acidic pH and high Ca^2+^ using two different combinations of mitochondrial substrate + inhibitor conditions. We utilized pyruvate + rotenone (ROT) to mimic abundance of pyruvate with impaired complex I, or succinate + AA to mimic abundance of succinate with impaired complex III (Turrens and Boveris, 1980; Kakinuma et al., 1994; Starkov et al., 2004). The elevation in Ca^2+^ was to induce mitochondrial matrix Ca^2+^ overload sufficient to induce mPTP opening to mimic an effect of cardiac IR injury. We hypothesized that a decrease in pH, to further mimic conditions of ischemia, would additionally modulate O^•−^_2_ generation from complex III and/or complex I as assessed by H_2_O_2_ release in isolated mitochondria.

Mitochondrial inhibitors of complexes I-V can cause either reduced or enhanced O^•−^_2_ generation depending on their site of action (Becker et al., 1999). With pyruvate as the substrate, we chose ROT because it inhibits transfer of electrons from iron-sulfur (Fe-S) centers in complex I at the binding site for quinol, thus creating a backup of electrons and a highly reduced NADH pool. This scenario would mimic IR-induced damage to complex I proteins to cause impaired electron transfer via Fe-S centers (Gadicherla et al., 2012). Studies have shown that selective accumulation of succinate is a universal metabolic signature of ischemia in the heart and is responsible for mitochondrial ROS production during reperfusion (Lukyanova, 2013; Chouchani et al., 2014). Therefore, with succinate as the substrate, we chose AA because it inhibits the quinone -reducing center (Q_i_) of complex III to prevent the semiquinone radical formed at the Q_o_ site from being oxidized. Since this impedes electron transfer to the Q_i_ site, the semiquinone can then transfer its singlet electron to O_2_ to produce O^•−^_2_ at complex III (Starkov and Fiskum, 2001). With succinate, AA can also enhance O^•−^_2_ generation at complex I. This scenario mimics damage to complex I plus damage to the Fe-S peptide of complex III during ischemia when quinol oxidation at the Q_o_ site is limited; this leads to “bypass reactions” that enhance O^•−^_2_ generation at that site (Lesnefsky et al., 2001; Muller et al., 2002). We chose not to block complex IV, which would mimic diminished O_2_ levels during ischemia, because in this case it would prevent O^•−^_2_ generation at complex III like the complex III Q_o_ site inhibitor myxothiazol, while stimulating O^•−^_2_ generation at complex I (Turrens et al., 1985).

## Materials and methods

All experiments were performed in accordance with the National Institutes of Health (NIH) Guide for the Care and Use of Laboratory Animals (NIH Publication No. 85–23, revised 1996) and were approved by the Institutional Animal Care and Use Committee of the Medical College of Wisconsin.

### Mitochondria isolation

Heart mitochondria were isolated from ketamine-anesthetized (50 mg/kg ip) guinea pigs (250–350 g) as described previously (Gadicherla et al., 2012; Aldakkak et al., 2013; Blomeyer et al., 2013). Briefly, ventricles were excised, placed in an isolation buffer (buffer A) that contained (in mM) 200 mannitol, 50 sucrose, 5 KH_2_PO_4_, 5 MOPS, 1 EGTA, and 0.1% bovine serum albumin (BSA; all chemicals from Sigma, St. Louis, MO, USA), with pH adjusted to 7.15 with KOH. Ventricles were then minced into 1-mm^3^ pieces. The suspension was homogenized in isolation buffer containing 5U/ml protease (Bacillus licheniformis; Sigma), followed by differential centrifugation at 4°C, and the final pellet was resuspended in isolation buffer and kept on ice. Protein content was determined by the Bradford method. Mitochondrial suspension was adjusted to yield 12.5 mg protein/ml for experimental purpose. Details of the experimental approach are provided in the Supplementary Material.

### Experimental protocol

Experiments were conducted at room temperature (25°C), with mitochondria (0.5 mg protein/ml) suspended in experimental buffer (buffer B) that contained (in mM) 130 KCl (EMD Chemicals, Gibbs-town, NJ, USA), 5 K_2_HPO_4_, 20 MOPS, 0.001 Na_4_P_2_O_7_, and 0.1% BSA. This assured that only 40 μM EGTA was carried over from the isolation buffer (buffer A) into the experimental buffer. Based on the experimental protocol and conditions, the buffer pH was specifically adjusted upward from 6.5 to 6.9 and 7.15 by adding KOH. The respiration buffer contained 0 or 150 μM CaCl_2_; concentrations of CaCl_2_ between 20 and 60 μM had no significant effects on H_2_O_2_ production (Figure S.3) and 100 μM CaCl_2_ gave inconsistent data, so these data are not reported. From the residual EGTA concentration of 40 μM, we estimated 150 μM CaCl_2_ to be equivalent to ≈ 220 nmol CaCl_2_/mg protein. After adding CaCl_2_ (or H_2_O), 10 mM Na^+^ pyruvate or Na^+^ succinate (Sigma) was added. Then either complex I blocker ROT (10 μM; Sigma) or complex III blocker AA (5 μM; Sigma) was added.

### Mitochondrial fluorescence measurements

Mitochondria were suspended in buffer B in a 1 ml cuvette inside a spectrophotometer (QM-8; Photon Technology International (PTI), Birmingham, NJ, USA). The rate of H_2_O_2_ release was measured using Amplex red (12.5 μM; Molecular Probes, Eugene, OR, USA) and horseradish peroxidase (0.1 U/ml; Sigma) at excitation and emission wavelengths of 530 and 583 nm, respectively. H_2_O_2_ is the direct product of O^•−^_2_ when catalyzed by O^•−^_2_ dismutase (SOD) in the absence of nitric oxide. H_2_O_2_ levels were calibrated over a range of 10–200 nM H_2_O_2_ (Sigma) added to buffer B in the absence of mitochondria and in the presence of Amplex red and horseradish peroxidase. Mitochondrial volume change (increase/decrease) was assessed by monitoring changes in 90° light scattering at an excitation and emission wavelength of 520 nm inside the same cuvette-based PTI.

### Mitochondrial O_2_ consumption

Oxygen consumption was measured polarographically using a respirometry system (System S 200A; Strathkelvin Instruments, Glasgow, Scotland). Respiration experiments using pyruvate or succinate at pH 7.15 and without Ca^2+^ were initially conducted to determine the viability of mitochondria for the rest of the experiments. Respiration was initiated by adding 10 mM complex I substrate Na^+^ pyruvate or the complex II substrate Na^+^ succinate. State 3 respiration was measured after adding 250 μM ADP (Sigma), and state 4 respiration was measured after complete phosphorylation of the added ADP. The respiratory control index (RCI) was calculated as the ratio of the rate of state 3 to state 4 respiration. Only mitochondria with an RCI of 10 or above with pyruvate or an RCI of 3 or above with succinate were used in the experiments. To assess the effects of pH and extra-matrix (e) [Ca^2+^]_e_ on O_2_ consumption (respiration), we added either H_2_O (control) or CaCl_2_ for a final concentration of 150 μM to the mitochondrial suspension at three pHs (7.15, 6.9 or 6.5) before adding substrates.

## Results

### Effect of changing buffer pH and Ca^2+^ on mitochondrial release of H_2_O_2_ and volume after inhibiting complex I in pyruvate-energized mitochondria

**Figure 1:**
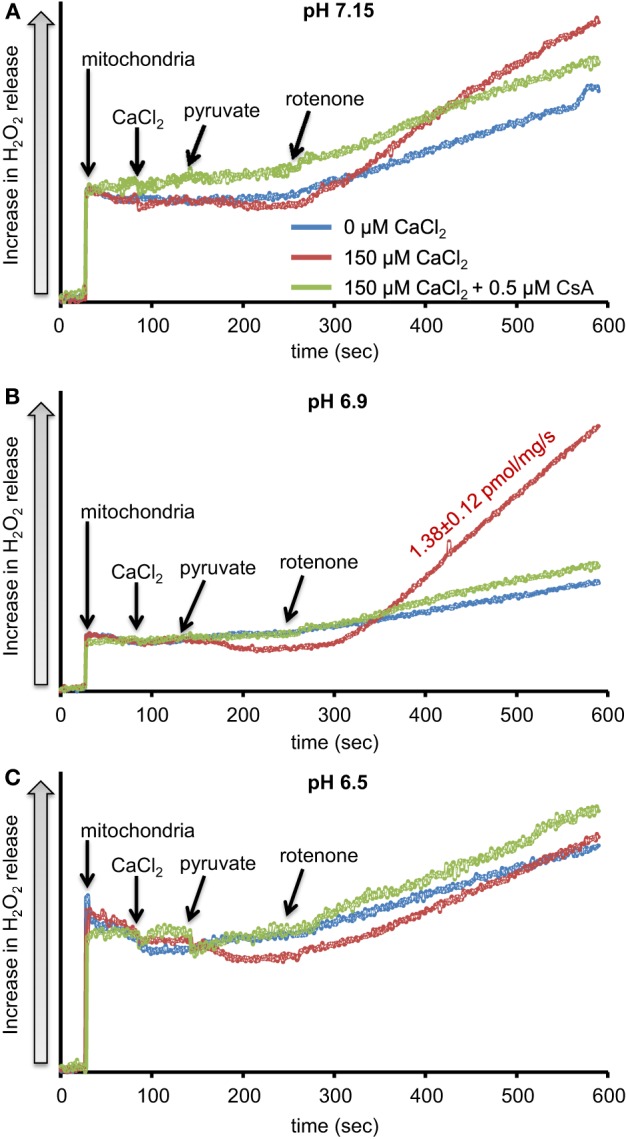
**Time-dependent changes in H_2_O_2_ release rates in isolated pyruvate-energized mitochondria after inhibiting complex I with rotenone (ROT) at a different pH: (A) pH 7.15, (B) pH 6.9, (C) pH 6.5**. Mitochondria were added to buffer at 30 s, CaCl_2_ at 90 s, pyruvate at 150 s, and ROT at 270 s. The blue trace represents no added CaCl_2_, the red trace represents added CaCl_2_to 150 μM in the presence of 40 μM EGTA (≈ 220 nmol/mg free Ca^2+^), and the green trace represents added CaCl_2_ to 150 μM with cyclosporine A (CsA). H_2_O_2_ release was assessed using amplex red with horseradish peroxidase. Numbers indicate mean values ± SEM of pmol H_2_O_2_generated/mg/s. *N* = 4 each.

**Figure 2:**
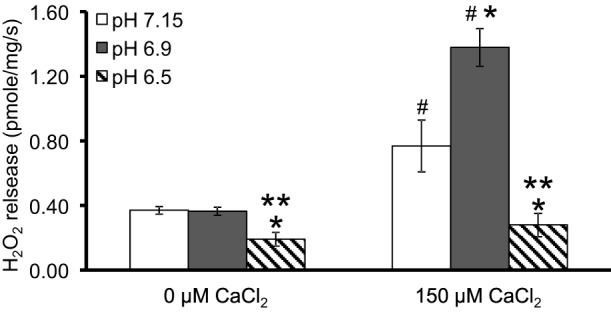
**Summary of the effects of pH and added CaCl_2_ on H_2_O_2_ release rates in pyruvate-energized mitochondria after inhibiting complex I with ROT**. Columns represent mean values ± SEM of pmol H_2_O_2_ emission/mg/s. *P* < 0.05 ^*^Significant difference in H_2_O_2_ release rate at pH 6.9 or pH 6.5 vs. pH 7.15 within the same CaCl_2_ group. ^**^Significant difference in H_2_O_2_ release rate at pH 6.5 vs. pH 6.9 within the same CaCl_2_ group. ^#^Significant difference in H_2_O_2_ release rate in 150 μM CaCl_2_ vs. 0 μM CaCl_2_ for each pH group. *N* = 4 each.

**Figure 3:**
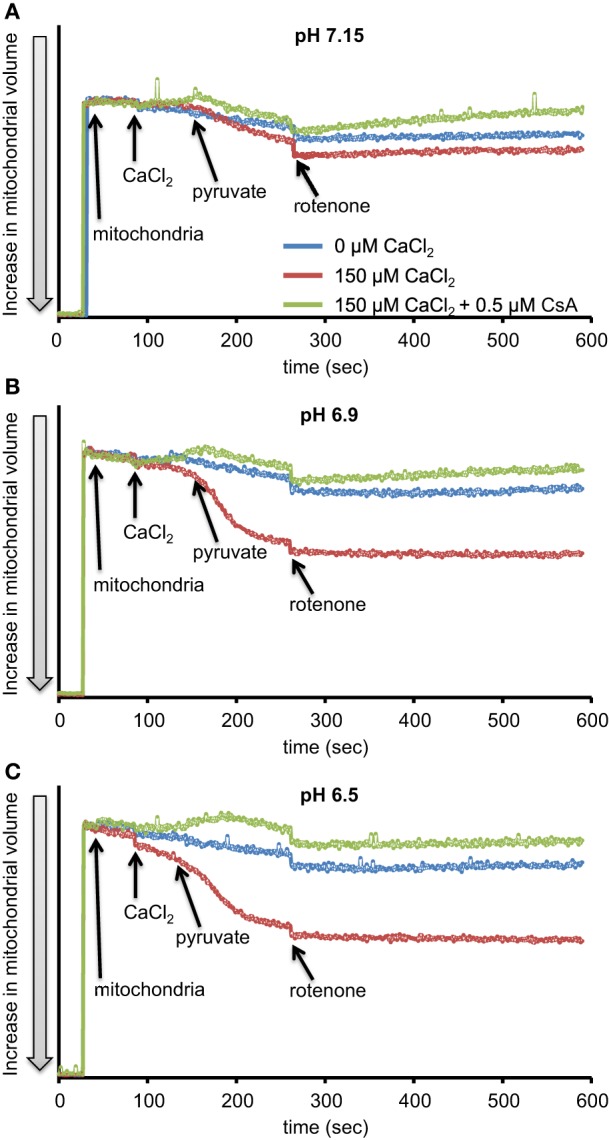
**Time-dependent changes in mitochondrial volume in isolated pyruvate-energized mitochondria after inhibiting complex I with ROT at a different pH: (A) pH 7.15, (B) pH 6.9, (C) pH 6.5**. Mitochondria were added to buffer at 30 s, CaCl_2_ at 90 s, pyruvate at 150 s, and ROT at 270 s. The blue trace represents no added CaCl_2_, the red trace represents added CaCl_2_ to 150 μM in the presence of 40 μM EGTA (≈ 220 nmol/mg free Ca^2+^), and the green trace represents added CaCl_2_to 150 μM with cyclosporine A (CsA). Mitochondrial volume was assessed by measuring changes in 90° light scattering. *N* = 3 each.

We first evaluated H_2_O_2_ release rates resulting from ROT-inhibited complex I in pyruvate-energized mitochondria at pHs 7.15, 6.9, and 6.5, each with H_2_O (control) or with added 150 μM CaCl_2_ (Figures 1A–C, 2). In the absence of CaCl_2_, adding ROT caused a modest increase in H_2_O_2_ release rate at each pH. In the presence of 150 μM CaCl_2_, adding ROT caused a marked increase in H_2_O_2_ release rate at pH 6.9 (1.38 ± 0.12 pmol/mg/s) compared to pH 7.15 (0.37 ± 0.02 pmol/mg/s) or to pH 6.5 (0.28 ± 0.07 pmol/mg/s). Since high Ca^2+^ is known to induce mPTP opening (Camara et al., 2010), we tested the hypothesis that the Ca^2+^-induced H_2_O_2_ increase is a result of mPTP opening. Addition of cyclosporine A (CsA; 0.5 μM; Sigma) prevented the large increase in H_2_O_2_ release, which was more apparent at pH 6.9 (0.39 ± 0.04 pmol/mg/s). To further evaluate the role of mPTP, we measured the corresponding mitochondrial volume changes in pyruvate-energized mitochondria in the same combinations of buffer pH and CaCl_2_ with later addition of ROT (Figures 3A–C). Adding CaCl_2_ alone did not significantly alter mitochondrial volume before addition of pyruvate. However, at pH 6.9 (Figure 3B) and 6.5 (Figure 3C), mitochondrial volume significantly increased with added 150 μM CaCl_2_ after adding pyruvate, but volume was not significantly affected at pH 7.15 (Figure 3A). Additionally, mitochondrial volume did not change significantly in experiments without added CaCl_2_. Adding CsA prevented the large increases in mitochondrial volume; adding ROT stopped any increases in volume (Figures 3A–C). The effect of adding superoxide SOD on H_2_O_2_ release under these conditions is given in Supplementary Materials.

### Effect of changing buffer pH and Ca^2+^ on mitochondrial release of H_2_O_2_ and volume after inhibiting complex III in succinate-energized mitochondria

**Figure 4:**
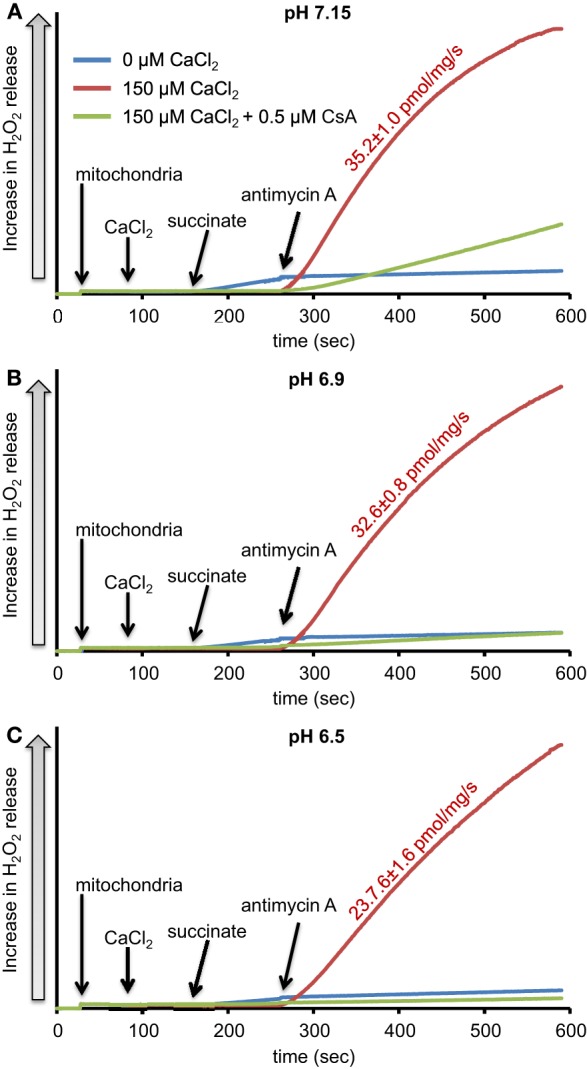
**Time-dependent changes in H_2_O_2_ release rates in isolated succinate-energized mitochondria after inhibiting complex III with antimycin A (AA) at a different pH: (A) pH 7.15, (B) pH 6.9, (C) pH 6.5**. Mitochondria were added to buffer at 30 s, CaCl_2_ at 90 s, succinate at 150 s, and AA at 270 s. The blue trace represents no added CaCl_2_, the red trace represents added CaCl_2_to 150 μM in the presence of 40 μM EGTA (≈ 220 nmol/mg free Ca^2+^), and the green trace represents added CaCl_2_to 150 μM with CsA. Numbers indicate mean values ± SEM of pmol H_2_O_2_generated/mg/s. *N* = 4 each.

**Figure 5:**
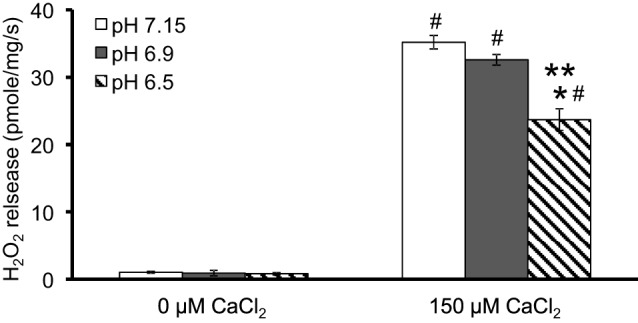
**Summary of the effects of pH and added CaCl_2_ on rates of H_2_O_2_ release rate in succinate-energized mitochondria after inhibiting complex III with AA**. Columns represent mean values ± SEM of pmol H_2_O_2_ generated/mg/s. *P* < 0.05 ^*^Significant difference in H_2_O_2_ release rate at pH 6.9 or pH 6.5 vs. pH 7.15 within the same CaCl_2_ group. ^**^Significant difference in H_2_O_2_ release rate at pH 6.5 vs. pH 6.9 within the same CaCl_2_ group. ^#^Significant difference in H_2_O_2_ release rate in 150 μM CaCl_2_ vs. 0 μM CaCl_2_ for each pH group. *N* = 4 each.

**Figure 6:**
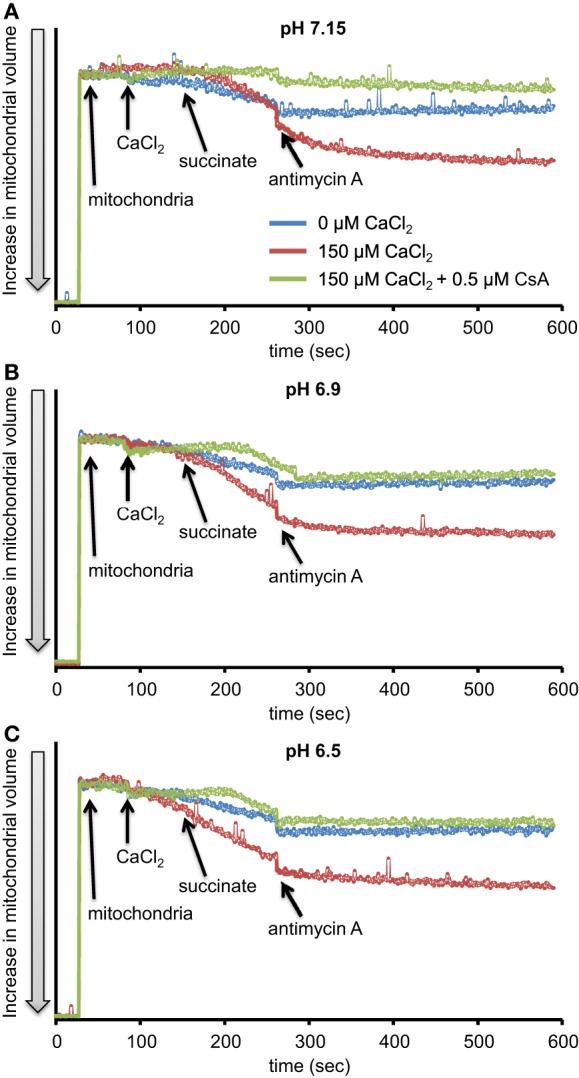
**Time-dependent changes in mitochondrial volume in isolated succinate-energized mitochondria after inhibiting complex III with AA at a different pH: (A) pH 7.15, (B) pH 6.9, (C) pH 6.5**. Mitochondria were added to buffer at 30 s, CaCl_2_ at 90 s, succinate at 150 s, and AA at 270 s. The blue trace represents no added CaCl_2_, the red trace represents added CaCl_2_to 150 μM in the presence of 40 μM EGTA (≈ 220 nmol/mg free Ca^2+^), and the green trace represents added CaCl_2_ with CsA. *N* = 3 each.

We evaluated H_2_O_2_ release rates resulting from adding AA to succinate-energized mitochondria at pH 7.15, 6.9, or 6.5, without (control) or with added 150 μM CaCl_2_ (Figures 4A–C, 5). In the absence of CaCl_2_ at all pHs tested, adding succinate prior to adding AA caused an increase in H_2_O_2_ release rate while later addition of AA reduced succinate-induced H_2_O_2_ release rate (Figures 4A–C). In the presence of 150 μM CaCl_2_ without AA, adding succinate did not significantly increase H_2_O_2_ release. However, later addition of AA with added CaCl_2_ (150 μM) caused a large increase in H_2_O_2_ release rate at all pHs with pH 7.15 showing the highest rate (35.2 ± 1.0 pmol/mg/s), followed by pH 6.9 (32.6 ± 0.8 pmol/mg/s), and then by pH 6.5 (23.7 ± 1.6 pmol/mg/s) (Figures 4A–C, 5). Adding CsA prevented the Ca^2+^-induced increase in H_2_O_2_ release rate resulting from AA treatment at each pH with the least effect on mPTP at pH 7.15. Because enzyme activity is pH dependent, we examined the effect of adding SOD to the buffer under the same experimental conditions. The effect of adding SOD on H_2_O_2_release was minimal (Figures S.1, S.2). In parallel, we measured the corresponding mitochondrial volume changes in succinate-energized mitochondria in the same combinations of pH and extra-matrix CaCl_2_ with later addition of AA (Figures 6A–C). Adding CaCl_2_ alone did not significantly alter mitochondrial volume before adding succinate (Figures 6A–C). Later addition of succinate induced a significant increase in mitochondrial volume at all pHs which was prevented by CsA. Adding AA led to an attenuation of mitochondrial volume (Figures 6A–C).

### Effect of changing buffer pH and Ca^2+^ on mitochondrial respiration in pyruvate and succinate-energized mitochondria

**Figure 7:**
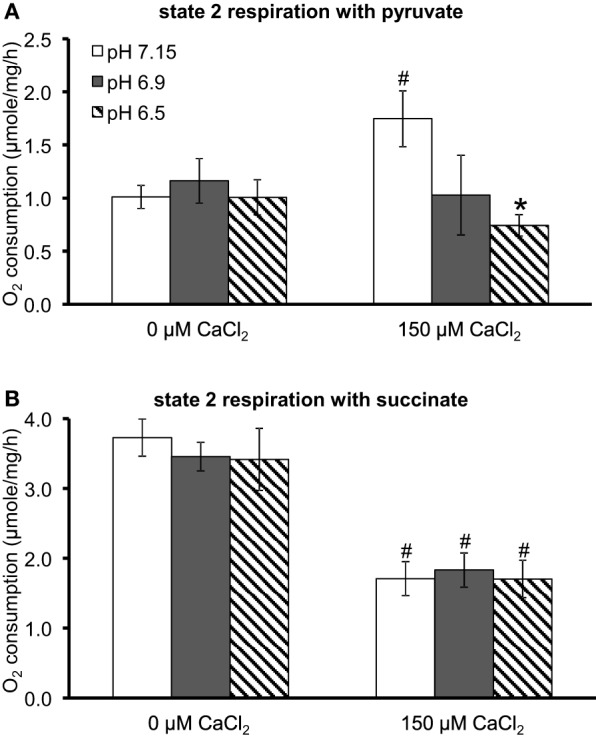
**Summary of the effects of pH and added CaCl_2_ on O_2_ consumption (μmol/mg/h) during state 2 respiration in (A) pyruvate-energized and (B) succinate-energized mitochondria**. *P* < 0.05 ^*^Significant difference in O_2_ consumption at pH 6.9 or pH 6.5 vs. pH 7.15 within the same CaCl_2_ group. ^#^Significant difference in O_2_ consumption in 150 μM CaCl_2_ vs. 0 μM CaCl_2_ for each pH group. O_2_ consumption was measured in a respirometer. *N* = 4 each.

Because O^•−^_2_ generation is dependent on electron flux through the ETC and is a product of electron leak at several complexes, we evaluated the effects of substrate, pH, and [Ca^2+^]_e_ on mitochondrial respiration during states 2, 3, and 4 respiration (all of the above experiments were conducted during state 2). In pyruvate-energized mitochondria (Figure 7A), and before adding CaCl_2_, there was no difference in state 2 respiration among all pH groups. Adding 150 μM CaCl_2_ led to an increase in state 2 in the pH 7.15 group only. In succinate-energized mitochondria (Figure 7B), and before adding CaCl_2_, there was no difference in state 2 respiration among all pH groups. However, adding 150 μM CaCl_2_ led to similar decreases in state 2 respiration for all pH groups. States 3 and 4 respiration rates and the respective RCI values for each pH group without (control) or with addition of 150 μM CaCl_2_ are summarized (Table 1). Adding 150 μM CaCl_2_ decreased states 3 and 4 respiration and RCI under each of the two substrate and three pH conditions, except at pH 7.15 for state 4 respiration with pyruvate where state 4 respiration increased slightly but significantly. State 3 respiration and RCI were unaffected by pH except in two conditions: (1) pyruvate and 0 μM CaCl_2_, in which they were reduced at pH 6.5, and (2) pyruvate and 150 μM CaCl_2_, in which they were reduced at pH 6.9 and 6.5. State 4 respiration was unaffected by pH except in the condition pyruvate and 150 μM CaCl_2_, in which it was reduced at pH 6.9 and 6.5.

**Table 1:** **Effects of increasing concentrations of CaCl_2_ on states 3, and 4, and respiratory control index (RCI, state 3/state 4) under different substrate and pH conditions**.

	**Substrate**	**μM CaCl_2_**	**pH 7.15**	**pH 6.9**	**pH 6.5**
State 3	Pyruvate	0	13.7 ± 0.8	14.6 ± 0.9	9.2 ± 1.3^*^
		150	9.0 ± 1.0^‡^	0.9 ± 0.4^*^^‡^	0.5 ± 0.1^*^^‡^
State 3	Succinate	0	11.4 ± 1.1	12.5 ± 0.5	9.9 ± 3.9
		150	2.0 ± 0.2^‡^	1.9 ± 0.3^‡^	1.9 ± 0.3^‡^
State 4	Pyruvate	0	1.1 ± 0.1	1.2 ± 0.1	1.3 ± 0.2
		150	2.3 ± 0.2^‡^	0.7 ± 0.3^*^^‡^	0.6 ± 0.2^*^^‡^
State 4	Succinate	0	3.9 ± 0.4	3.7 ± 0.3	3.2 ± 0.5
		150	2.4 ± 0.3^‡^	2.3 ± 0.3^‡^	2.1 ± 0.3^‡^
RCI (state 3/state 4)	Pyruvate	0	12.6 ± 0.7	12.0 ± 0.4	7.2 ± 0.6^*^^§^
		150	4.0 ± 1.1^‡^	1.3 ± 0.3^*^^‡^	1.0 ± 0.1^*^^‡^
RCI (state 3/state 4)	Succinate	0	3.1 ± 0.5	3.8 ± 0.4	3.5 ± 0.6
		150	0.9 ± 0^‡^	0.9 ± 0^‡^	0.9 ± 0.0^‡^

P < 0.05

* *Significant difference in states 3 and 4 and RCI in pH 6.9 or pH 6.5 vs. pH 7.15 within the same CaCl_2_ group*.

§ *Significant difference in RCI in pH 6.5 vs. pH 6.9 within the same CaCl_2_ group*.

‡ *Significant difference in states 3 and 4 and RCI in 150 μM CaCl_2_ vs. 0 μM CaCl_2_ for each pH group. Note the marked effect of 150 μM CaCl_2_ to depress RCI at each pH and the smaller effect of pH 6.5 with pyruvate and no CaCl_2_ to reduce RCI*.

## Discussion

The main goal of this work was to simulate in isolated heart mitochondria prevailing effects that can occur in cardiac IR injury such as excess Ca^2+^, low pH, and impaired electron transfer at ETC complexes I and III, and to determine their impact on release of H_2_O_2_. In addition, we used either the complex I substrate pyruvate or the complex II substrate succinate to mimic substrate conditions that may prevail during IR. The first protocol (e.g., Figure 3) was intended to represent less severe IR injury (pyruvate as substrate, inhibited complex I > complex III, low Ca^2+^ loading, pH 7.15, 6.9); the second protocol was intended to represent more severe IR injury (succinate as substrate, inhibited complex III + complex I, high Ca^2+^ loading, pH 6.5). Under these latter conditions, H_2_O_2_ release rate was about 10 times higher, and the lower pH with either substrate condition attenuated H_2_O_2_ release. Overall, our results show that in succinate energized mitochondria, increased buffer Ca^2+^ enhances mitochondrial H_2_O_2_ release rates resulting from complex I and III (AA) similarly at each pH, whereas in pyruvate energized mitochondria, a significant increase in H_2_O_2_ release rate resulting from complex I (ROT) occurs only at pH 6.9.

### Complex I and complex III are primary sources of O^•−^_2_ during ischemia and reperfusion

It is well-documented that reperfusion after ischemia is associated with considerable ROS emission (Vanden Hoek et al., 1997; Becker et al., 1999; Kevin et al., 2003). It appears paradoxical that ROS are also produced during ischemia; but total mitochondrial anoxia is unlikely to exist even with extensive cardiac ischemia (Becker, 2004). It has been reported that isolated mitochondria produce H_2_O_2_ when O_2_ is as low as 0.5 mM (*p*O_2_ of about 10 mmHg) (Saborido et al., 2005). Mitochondrial *p*O_2_ *in vivo* is only about 1–5 mmHg with normoxia, so mitochondria normally thrive in a low O_2_ environment. The emission of ROS is due to excessive O^•−^_2_ generation (likely during early mild ischemia when the redox potential is high) and to diminished ROS scavenging (likely during later severe ischemia when the redox potential is low).

In previous studies of IR injury in guinea pig isolated hearts, we observed a modest increase in O^•−^_2_ generation during early ischemia (10–15 min) followed by a larger increase in O^•−^_2_ generation during late ischemia (20–30 min) and a surge during early reperfusion (Kevin et al., 2003; Riess et al., 2004; Aldakkak et al., 2008a,b, 2011). Recently, we demonstrated in isolated mitochondria, under conditions simulating ischemia, that CaCl_2_ addition in the presence of succinate resulted in enhanced H_2_O_2_ release when complex III was blocked (Aldakkak et al., 2013).

It is widely recognized that both mitochondrial complexes I and III play a crucial role in producing ROS during cardiac IR injury, particularly with induction of mPTP. “Triggering” amounts of ROS can be generated in isolated cardiomyocytes by photoactivation of tetramethylrhodamine derivatives that leads to membrane depolarization (mPTP induction) and a burst of ROS, which is coined “ROS-induced ROS release (RIRR)” (Zorov et al., 2000). However, inducing mPTP opening with excess CaCl_2_ in isolated liver mitochondria did not increase H_2_O_2_ release unless exogenous NADH was added to the buffer (Batandier et al., 2004). Because mPTP opening resulted in a ROT-sensitive impairment of complex I activity, they concluded that mPTP opening is associated with defective electron transfer within complex I, leading to O^•−^_2_ release at that site. We did not add NADH to the buffer but we observed an increase in H_2_O_2_ release under either substrate condition when complex I and III inhibitors were used to mimic some conditions of IR injury. Despite these advances, the prevailing metabolic conditions during mild vs. severe IR injury that promote differential dysfunction of the complexes to cause excess O^•−^_2_ generation and ROS emission in a vicious cycle of RIRR remain unclear.

In ischemia, decreasing O_2_ levels and concomitant activation of glycolysis caused a decrease in pH, increased extra-matrix and matrix [Ca^2+^], and eventually damage to complexes I and III due to oxidative stress (Rouslin, 1983). O^•−^_2_ generated under these conditions is derived in part from complex I through forward electron transfer (FET) (Starkov et al., 2004). In the study above (Rouslin, 1983), it was reported that the activity of complex I decreased markedly after 20 min ischemia and that this decrease closely paralleled the decrease in mitochondrial O_2_ uptake with NADH-linked substrates; it was also reported that the activity of complex III decreased at a more gradual rate during ischemia and that its rate of decrease paralleled that of succinate-supported O_2_ uptake. As ischemia progresses, pH drops further and extra-matrix and matrix Ca^2+^ levels rise, while substrate utilization switches from primarily pyruvate to mostly succinate, which accumulates from 0.2 to 0.4 mM during normoxia to 4–7 mM during ischemia or hypoxia (Kakinuma et al., 1994; Starkov et al., 2004). Based on these studies, we used pyruvate + ROT to approximate the condition of impaired electron transfer via complex I to complex III, and succinate + AA to approximate the condition of enhanced utilization of succinate at complex II and impaired electron transfer through complex I (by RET) and III. Our protocols were also based on a summary of data (Lukyanova, 2013) stating that the switch from normoxia to hypoxia increased succinate utilization by complex II from about 25–35% to 65–85% while complex I activity was mostly inhibited. Under this condition, complex III appears to be damaged by ROS due to the initial O^•−^_2_ generated at complex I, which is analogous to AA inhibition of complex III (Musatov and Robinson, 2012). Given these conditions and the diminished ΔΨ_m_, ROS production during severe IR injury likely results from FET including complex II and III. Therefore, our experiments with succinate + AA may mimic the conditions of O^•−^_2_ generated at both complex I and III during IR injury.

### Biphasic effects of pH on mPTP leads to varying H_2_O_2_ production from complex I

Adding CaCl_2_ and lowering pH both modulated H_2_O_2_ production in pyruvate-energized mitochondria after adding ROT (Figures 1A–C, 2). Based on previous studies showing a distinct early phase of H_2_O_2_ production during mild ischemia correlated with the timing of complex I damage, we hypothesized that pyruvate-energized mitochondria with inhibited complex I and added CaCl_2_ would show an increased H_2_O_2_ release rate as buffer pH decreased. Indeed, a sharp rise in H_2_O_2_ production was observed at pH 6.9 on addition of CaCl_2_, which was not seen at pH 7.15. However, as pH decreased further to 6.5, a rise in H_2_O_2_ did not occur. It is unclear what the exact mechanism is for the large increase in H_2_O_2_ release in pyruvate-supported mitochondria at pH 6.9 with added CaCl_2_. It is important to note that under these conditions, there was a significant increase in mitochondrial volume, probably indicating mPTP opening. Indeed, addition of CsA to desensitize mPTP prevented the increase in mitochondrial volume and reduced H_2_O_2_ release to levels similar to those observed at pH 7.15. Nonetheless, mPTP opening occurred also at pH 6.5 with high CaCl_2_ as indicated by the increase in mitochondrial volume that was prevented with CsA; but this was not associated with a large increase in H_2_O_2_ release.

mPTP opening can occur during mitochondrial Ca^2+^ overload (Orrenius et al., 2003), whereas a low mitochondrial pH is associated with a reduced probability of mPTP opening. For example, reoxygenation or reperfusion under acidic conditions is associated with much lower ROS emission (Haworth and Hunter, 1979; Halestrap, 1991; Bernardi et al., 1992). However, a previous study (Halestrap, 1991), with glutamate/malate-energized mitochondria isolated from rat hearts, showed a graded effect of pH on the probability of mPTP opening with the least likelihood of opening at pH 6.0 and below. At pHs above 6.0 there was a significant increase in the probability of mPTP opening. Indeed, at pH 6.5, a Ca^2+^ -induced increase in volume, presumably through mPTP opening, was demonstrated to be greater than 40% of the increase seen at pH 7.4; and pH 6.9 had approximately 75% of the volume increase seen at pH 7.4 (Halestrap, 1991).

mPTP opening is proposed to increase ROS emission through three mechanisms: a loss of glutathione leading to decreased ROS scavenging, a loss of cytochrome *c* leading to increased reduction of upstream ETC complexes and subsequent electron loss and diminished scavenging, and an increase in ROS derived from the α-ketoglutarate dehydrogenase complex due to loss of NAD^+^ (Camara et al., 2010, 2011; Toledo et al., 2014). In our study different probabilities of mPTP opening at pH 6.9 and 6.5 may be responsible for the disparity in H_2_O_2_ levels generated at these two pHs. Limited opening of mPTP at pH 6.5 may be responsible for H_2_O_2_ generation at levels not sufficient to induce RIRR, whereas at pH 6.9 the increased opening of mPTP may induce RIRR. Additionally, the lack of increased H_2_O_2_ generation and mitochondrial volume at pH 7.15, when compared to pH 6.9, might be related to an increase in inhibition of complex I with high CaCl_2_ as pH increases (Sadek et al., 2004; Chen et al., 2010). The decrease in complex I activity leading to decreased ROS production might in turn prevent RIRR and subsequent opening of the mPTP (Zorov et al., 2006). Thus, the combined effects of inhibited complex I activity and mPTP opening at pH 6.9 might explain the elevation in H_2_O_2_ release rates observed at pH 6.9, but not at pHs 7.15 and 6.5.

### pH-dependent O^•−^_2_ generation from complexes I and III and stability of semiquinone radical

Adding CaCl_2_ and altering pH also modulated H_2_O_2_ generation in succinate-energized mitochondria with added AA (Figures 4A–C). With AA, mitochondria in buffer with high CaCl_2_ showed a dramatic rise in H_2_O_2_ release at all pHs. The increase in H_2_O_2_ corresponded to an increase in pH. In addition, an increase in mitochondrial volume occurred at each pH in the presence of high CaCl_2_, and these conditions led to increased H_2_O_2_ release, suggesting a role for mPTP opening. Indeed, both the increases in H_2_O_2_ release and volume were inhibited by adding CsA at each pH. H_2_O_2_ generation under these conditions is likely caused by FET from complex II through complex III, because adding AA decreased ΔΨ_m_, which would prevent RET from occurring. In this case, the primary source of H_2_O_2_ is that derived from O^•−^_2_ generated at the Q_o_ site of complex III.

Mitochondrial pH may have a significant role in moderating O^•−^_2_ generation by complex III. Matrix alkalinization (higher pH) tended to stabilize the semiquinone radical at the Q_o_ site (Selivanov et al., 2008). This was proposed to result from decreased binding of H^+^ ions necessary to drive the Q cycle forward (Selivanov et al., 2008). Stability of the semiquinone radical leads to increased likelihood of direct transfer of an electron to an O_2_ molecule, leading to the formation of O^•−^_2_ (Selivanov et al., 2008). Additionally, because AA blocks the oxidation of semiquinone at Q_i_ and the transfer of an electron from the Q_o_ site, this can lead to increased O^•−^_2_ generation from the Q_o_ site, which may be analogous to impaired complex III function during ischemia (Chen et al., 2008; Musatov and Robinson, 2012). Consequently, in our experiments, the increased H_2_O_2_ release rate at a high pH is possibly related to the increased stability of semiquinone leading to increased direct electron donation to O_2_ to generate O^•−^_2_.

### Ca^2+^ -induced mPTP opening and mitochondrial respiration

Mitochondrial uncouplers like dinitrophenol tend to increase respiration to counteract a decline in ΔΨ_m_ due to H^+^ leak. But others have reported that it is not unusual for mitochondrial uncouplers or uncoupling events such as mPTP opening to inhibit succinate-supported state 2 respiration. Mitochondrial uncouplers can retard succinate oxidation under some conditions (Papa et al., 1969). In the absence of ROT, and with succinate in high concentrations (conditions similar to those used in our study), mitochondrial uncouplers have been found to inhibit succinate oxidation due to the formation of oxaloacetate (Wojtczak et al., 1969; Vik and Hatefi, 1981; Kotlyar and Vinogradov, 1984; Drose, 2013).

In our study, the lower state 2 respiration after adding CaCl_2_ with succinate (Figure 7B) at each pH, may be due to a greater collapse in ΔΨ_m_ due to the excess influx of Ca^2+^. In contrast, the higher state 2 respiration after adding CaCl_2_ with pyruvate (Figure 7A) at pH 7.15 may be a result of enhanced H^+^ pumping at complex I by this NADH-linked substrate; however, as the trans-membrane pH potential is increased (and thus the proton motive force), a faster respiration might not be needed to maintain ΔΨ_m_. The RCI for pyruvate was reduced at pH 6.5 likely because of an uncoupling effect due to H^+^ leak with slower ATP production (Table 1). The RCI for succinate, which is much lower than that for pyruvate, appears to stem from the much higher basal respiratory rate for succinate vs. pyruvate (Figures 7B vs. 7A). In the absence of CaCl_2_, mitochondria showed well-coupled oxidative phosphorylation with both substrates at each pH, except in pyruvate-energized mitochondria at pH 6.5, in which case the RCI was lower, indicating relatively less coupling. The effect of added CaCl_2_ on states 3 and 4 respirations and RCI with either substrate or at any pH is likely a result of marked uncoupling due to mPTP opening (increased mitochondrial volume) because this was sensitive to CsA (Figures 3, 6).

### Summary, conclusion, and perspective

In conclusion, in our previous studies using the isolated, beating heart model of 30 min global ischemia, we demonstrated two phases of increased O^•−^_2_ generation, an early phase (10–20 min) that emits low to moderate O^•−^_2_ levels and a late phase (20–30 min) that emits higher O^•−^_2_ levels just before a surge in O^•−^_2_ release at the beginning of reperfusion. The present study sheds novel insights into the modulatory effect of matrix pH in Ca^2+^-induced mitochondrial H_2_O_2_ release. The early or mild phase of H_2_O_2_ release due to O^•−^_2_ generation at complex I could be related to differential effects of pH on the mPTP, which allows H_2_O_2_ production at the pH observed during early or mild ischemia (pH 6.9) but not at the lower or higher pHs. The late or severe phase of H_2_O_2_ release due to O^•−^_2_ generation primarily at complex III, but also at complex I, may also be dependent on mPTP opening, but H_2_O_2_ production is intensified with increasing pH. Therefore, it is possible that the surge in H_2_O_2_ production commonly observed on reperfusion results from O^•−^_2_ generated from complex III as the pH rises gradually with mPTP opening. Although both complex I and III contribute simultaneously to H_2_O_2_ production during IR, our results suggest that the role of each respiratory complex is not static but rather changes dynamically as the pH changes. Thus, each complex may play a more prominent role during a certain period of IR. Being cognizant of this information is important as it can be used to reduce ROS emission as ischemia progresses by targeting each complex separately, or possibly by manipulating the pH using Na^+^/Ca^2+^ and/or Na^+^/H^+^ exchange inhibitors, e.g., by maintaining a more alkaline environment during early ischemia and a more acidic environment during late ischemia/early reperfusion, to reduce O^•−^_2_ generation at complex I and III.

## Potential imitations

Although we have attempted to simulate some of the conditions in mitochondria that may occur during authentic cardiac IR injury, there are several shortcomings to this approach: (1) Our experimental design did not allow us to mimic the timing of ROS production during ischemia, or during reperfusion after ischemia, or to allow for the possible redox conditions associated with varying ROS scavenging capacity during IR injury. (2) We completely blocked electron transfer sites using inhibitors; *in vivo* it is known that IR injury impairs electron transfer, but does not block it completely. (3) IR injury could impede electron transfer at other sites (e.g., myxothiazol prevents semiquinone formation at the Q_o_ site), which could inhibit O^•−^_2_ generation at that site, but stimulate it at another site. (4) On the other hand, IR injury may cause O^•−^_2_ generation at other sites not examined (e.g., the flavin site of complex I). (5) Free fatty acids are a normal substrate for mitochondria and they were absent in this study. (6) The use of succinate + AA cannot distinguish O^•−^_2_ generation from complex I vs. III without knowledge of the redox state and ΔΨ_m_ or the presence of ROT to prevent RET. (7) Changes in matrix pH and Ca^2+^ during IR injury may not arise solely due to changes in the cytosol, but rather in the matrix in response to bioenergetic dysfunction and possibly loss of mitochondrial buffering capacity. (8) It is very difficult to directly assess O^•−^_2_ generation in mitochondria; thus a variation in the redox potential during IR injury can lead to differential dismutation of O^•−^_2_ to H_2_O_2_, an additional factor not assessed in our simulated ischemia conditions.

## Author contributions

DL Conducted the experiments, analyzed data, wrote the first draft. AC Assisted in developing the design of the study. Made critical revisions of the manuscript in development and after review. DS Assisted in developing the design of the study. Made critical revisions of the manuscript and Supplementary Materials in development and during revisions. RL Conducted supplemental experiments and assisted in suggestions to improve the revised manuscript and Supplementary Materials. MA Developed the structure, argument, and design of the study. Made critical revisions of the manuscript.

### Conflict of interest statement

The authors declare that the research was conducted in the absence of any commercial or financial relationships that could be construed as a potential conflict of interest.
