# Supplementary material for: Differential effects of buffer pH on Ca2+-induced ROS emission with inhibited mitochondrial complexes I and III
Source: Front Physiol. 2015 Mar 10;6:58. doi: 10.3389/fphys.2015.00058 (PMC4354303; doi:10.3389/fphys.2015.00058)
Supplement: Supplementary file 1 [file DataSheet1.DOCX]

**ONLINE SUPPLEMENTAL MATERIAL**

**Differential effects of buffer pH on Ca^2+^-induced ROS emission with**

**inhibited mitochondrial complex I and III**

Daniel P. Lindsay,^1^ Amadou K.. Camara,^1,3^ David F. Stowe,^1,2,3,4,5^ Ryan Lubbe,^1^

Mohammed Aldakkak^1*^

^1^Department of Anesthesiology, The Medical College of Wisconsin, Milwaukee, WI, USA

^2^Department of Physiology, The Medical College of Wisconsin, Milwaukee, WI, USA

^3^Cardiovascular Research Center, The Medical College of Wisconsin, Milwaukee, WI, USA

^4^Department of Anesthesiology, VA Medical Center Research Service, Milwaukee, WI, USA

^5^Department of Biomedical Engineering, Marquette University, Milwaukee, WI, USA

**Introduction**

Additional experiments were conducted to determine if exogenous superoxide dismutase (SOD) activity, as assessed by H_2_O_2_ release rate, was affected by the changes in buffer conditions, especially buffer pH.

**Methods**

**Mitochondria isolation**

Heart mitochondria were isolated from ketamine-anesthetized (50 mg/kg i.p.) guinea pigs (250–350 g) as described previously ([Gadicherla et al., 2012](#_ENREF_18);[Aldakkak et al., 2013](#_ENREF_3);[Blomeyer et al., 2013](#_ENREF_9)). Briefly, ventricles were excised, placed in an isolation buffer (buffer A) containing (in mM) 200 mannitol, 50 sucrose, 5 KH_2_PO_4_, 5 MOPS, 1 EGTA, and 0.1% bovine serum albumin (BSA; all chemicals were from Sigma, St. Louis, MO, USA), with pH adjusted to 7.15 with KOH. Ventricles were then minced into 1-mm^3^ pieces. The suspension was homogenized in isolation buffer containing 5U/ml protease (Bacillus licheniformis; Sigma), followed by differential centrifugation at 4°C, and the final pellet was resuspended in isolation buffer and kept on ice. Protein content was determined by the Bradford method. Mitochondrial suspension volume was adjusted to yield 12.5 mg protein/ml for experimental purpose. Experiments were conducted at room temperature (25°C), with mitochondria (0.5 mg protein/ml) suspended in experimental buffer (buffer B) that contained (in mM) 130 KCl (EMD Chemicals, Gibbstown, NJ, USA), 5 K_2_HPO_4_, 20 MOPS, 0.001 Na_4_P_2_O_7_, and 0.1% BSA (all chemicals from Sigma). This assured that only 40 μM EGTA was carried over from the isolation buffer (buffer A). Based on the experimental protocol and conditions, the buffer pH was specifically adjusted upward from 6.5 to 7.0 and 7.15 by adding KOH.

**Experimental protocol**

Mitochondria were suspended in the respiration buffer (buffer B) to a final concentration of 0.5 mg/ml. The respiration buffer contained 0 or 150 μM CaCl_2_ (residual EGTA of 40 μM), and either 0 or 40 U/ml of exogenous superoxide dismutase (CuSOD). This was followed by addition of 10 mM Na^+^ pyruvate or Na^+^ succinate (Sigma). Then either the complex I blocker rotenone (ROT, 10 μM; Sigma) or the complex III blocker antimycin A (AA, 5 μM; Sigma) was added.

**Mitochondrial H_2_O_2_** **fluorescence measurements**

Mitochondria were suspended in buffer B in a 1 ml cuvette inside a spectrophotometer (QM-8; Photon Technology International (PTI), Birmingham, NJ, USA). The rate of H_2_O_2_ release was measured using Amplex red (12.5 μM; Molecular Probes, Eugene, OR, USA) and horseradish peroxidase (0.1 U/ml; Sigma) at excitation and emission wavelengths of 530 and 583 nm, respectively. H_2_O_2_ is the direct product of O_2_^•−^ when catalyzed by SOD in the absence of nitric oxide. H_2_O_2_ levels were calibrated over a range of 10–200 nM H_2_O_2_ (Sigma) added to buffer B in the absence of mitochondria and in the presence of Amplex red and horseradish peroxidase.

**Results**

****In the primary experiments there was no SOD in the buffer so our measurement of H_2_O_2_ release (without exogenous SOD) was due to dismutation of superoxide (O_2_^•−^) by endogenous matrix SOD; freely membrane permeable H_2_O_2_ was detected in the buffer after it was released from the mitochondria. In the supplementary experiments presented here exogenous SOD was added to the buffer. We observed the following:

**Fig. S.1:** Summary of the effects of pH and added CaCl_2_ on rates of H_2_O_2_ release rate in pyruvate-energized mitochondria after inhibiting complex I with rotenone **a**) without exogenous CuSOD and **b**) with exogenous CuSOD. Columns represent mean values ±SEM in pmol H_2_O_2_ emission/mg/s. *P* < 0.05 *Significant difference in H_2_O_2_ release rate at pH 6.9 or pH 6.5 *vs*. pH 7.15 within the same CaCl_2_ group. ^†^Significant difference in H_2_O_2_ release rate with SOD compared to no SOD (control) within the same CaCl_2_ group. ^‡^Significant difference in H_2_O_2_ release rate at 150 µM CaCl_2_ *vs*. 0 µM CaCl_2_ for each pH group. N=4 each.

**Fig. S.2:** Summary of the effects of pH and added CaCl_2_ on rates of H_2_O_2_ release in succinate-energized mitochondria after inhibiting complex III with antimycin A, **a**) without exogenous CuSOD, and **b**) with exogenous CuSOD. Columns represent mean values ±SEM in pmol H_2_O_2_ emission/mg/s. *P* < 0.05 *Significant difference in H_2_O_2_ release rate with SOD compared to no SOD (control) within the same CaCl_2_ group. ^†^Significant difference in H_2_O_2_ release rate at pH 6.9 *vs*. pH 6.5 within the same CaCl_2_ group. ^‡^Significant difference in H_2_O_2_ release rate at 150 µM CaCl_2_ *vs*. 0 µM CaCl_2_ for each pH group. N=4 each.

**Fig. S.3:** Summary of the effects of pH and various concentrations of added CaCl_2_ on rates of H_2_O_2_ release rate in **a**) pyruvate and **b**) succinate-energized mitochondria after inhibiting complex I or III with rotenone or antimycin A, respectively. Columns represent mean values ±SEM in pmol H_2_O_2_ emission/mg/s. *P* <0.05 *Significant difference in H_2_O_2_ release rate at pH 6.9 or pH 6.5 vs. pH 7.15 within the same CaCl_2_ group. ^†^Significant difference in H_2_O_2_ release rate at pH 6.5 vs. pH 6.9 within the same CaCl_2_ group. ^‡^Significant difference in H_2_O_2_ release rate at 150 µM CaCl_2_ vs. 0-60 µM CaCl_2_ for each pH group. N=4 each.

**Discussion**

a) Addition of SOD increased the detection of H_2_O_2_ for all groups (each pH, both substrates, with and without ETC inhibitors) (Fig. S.1b *vs*. S.1a and Fig. S.2b *vs*. S2a). We interpret this to mean that some O_2_^•−^ must have been released across the mitochondrial membrane (likely by complex III) that was then dismutated by the exogenous buffer SOD; the remainder (minus buffer SOD) was dismutated internally.

b) The relationship among the different pH groups with pyruvate + rotenone changed slightly in the presence of SOD (Fig. S.2a,b). Without SOD, H_2_O_2_ release was greater at pH 6.9 > pH 7.15 > pH 6.5. With the addition of SOD, H_2_O_2_ increased in the pH 7.15 group to a level similar to that in the pH 6.9 group, whereas it remained lower in the pH 6.5 group.

c) The relationship among the different pH groups with succinate + antimycin A did not change significantly (Fig. 2a,b). In the absence of SOD, H_2_O_2_ release in the succinate + antimycin A group was higher at pH 6.9 > pH 7.15 > pH 6.5. This observation did not change with addition of SOD.

d) There was very little effect of pyruvate or succinate as substrates on H_2_O_2_ release when added CaCl_2_ was less than 150 µM (Fig. S.3a,b).

These results add some complexity to the interpretation. Since SOD increased H_2_O_2_ in the pH 7.15 group with pyruvate + rotenone, one may assume that at this pH the endogenous SOD activity is less than that at pH 6.9. However, if this were the case, then we should have observed a similar effect at pH 7.15 when succinate was the substrate, i.e., H_2_O_2_ release should increase even more at pH 7.15 when mitochondria are incubated with succinate + antimycin A. We therefore conclude that while SOD activity may play a role in the differences in H_2_O_2_ release among the different pH groups, it cannot solely explain all our findings. The chemical matrix scavenger MnTBAP may be useful to enhance scavenging of O_2_^•−^ generated into the matrix to distinguish this from extra-matrix O_2_^•−^ scavenging.

Our study showed a differential effect of pH on H_2_O_2_ release under two different substrate conditions in the presence or absence of Ca^2+^. Another factor in our measurement of H_2_O_2_ could be a minor contribution of *spontaneous* dismutation of O_2_^•−^ to H_2_O_2_. Indeed, spontaneous dismutation of O_2_^•−^ is also pH dependent with the maximal rate occurring at pH 4.8. However, spontaneous dismutation in our study is minimal for the following reasons: 1) If spontaneous dismutation contributed significantly then one would expect more O_2_^•−^ and hence a higher H_2_O_2_ release rate at pH 6.5 > pH 6.9 > pH 7.15. However, in pyruvate-energized mitochondria we did not observe a higher H_2_O_2_ release rate at pH 6.5 *vs*. pH 6.9 or pH 7.15. Moreover, in succinate-energized mitochondria the increase in H_2_O_2_ release rate was inversely related to the change in pH. 2) The rate of spontaneous dismutation of superoxide is 2 × 10^5^ M^-1^ S^-1^. A comparison of the rate constants indicates that at a physiological pH, enzyme catalyzed (SOD) dismutation is10,000 times faster than spontaneous chemical dismutation.

**References**

Gadicherla, A.K., Stowe, D.F., Antholine, W.E., Yang, M., and Camara, A.K. (2012). Damage to mitochondrial complex I during cardiac ischemia reperfusion injury is reduced indirectly by anti-anginal drug ranolazine. *Biochim Biophys Acta* 1817**,** 419-429. doi: 10.1016/j.bbabio.2011.11.021.

Aldakkak, M., Stowe, D.F., Dash, R.K., and Camara, A.K. (2013). Mitochondrial handling of excess Ca^2+^ is substrate-dependent with implications for reactive oxygen species generation. *Free Radic Biol Med* 56**,** 193-203. doi: 10.1016/j.freeradbiomed.2012.09.020.

Blomeyer, C.A., Bazil, J.N., Stowe, D.F., Pradhan, R.K., Dash, R.K., and Camara, A.K. (2013). Dynamic buffering of mitochondrial Ca^2+^ during Ca^2+^ uptake and Na^+^-induced Ca^2+^ release. *J Bioenerg Biomembr* 45**,** 189-202. doi: 10.1007/s10863-012-9483-7.
